# Prevalence and Associated Factors of Nomophobia Amongst the General Population in Makkah Province and Al-Madinah Province, Saudi Arabia: An Analytical Cross-Sectional Study

**DOI:** 10.2174/0117450179311620240508105100

**Published:** 2024-05-31

**Authors:** Bashar W. Sheikh, Nahla H. Hariri, Muath A. Alqahtani, Abdulkarim A. Aljabri, Abdullah S. Eterji, Saud M. Almutawa, Rahaf M. Aljohani, Sultan A. Metair, Tala A. Rawas, Nizar S. Bawahab, Alaa J. Alhejaili

**Affiliations:** 1 College of Medicine, Umm Al-Qura University, Makkah 24375, Saudi Arabia; 2 Department of Community Medicine and Health Care for Pilgrims, Faculty of Medicine, Umm AlQura University, Makkah 24375, Saudi Arabia; 3 College of Medicine, Taibah University, Al-Madinah 42353, Saudi Arabia; 4 Department of General Surgery, King Faisal Hospital, Makkah 24236, Saudi Arabia; 5 Department of Family Sciences, Faculty of Human Sciences and Design, King Abdulaziz University, Jeddah 21589, Saudi Arabia

**Keywords:** Nomophobia, Smartphone addiction, Prevalence, Depression, Insomnia, Mobile devices

## Abstract

**Background:**

Nomophobia is a public health issue that involves the fear of being without a mobile phone. The study aimed to estimate the prevalence of nomophobia and its relation to psychological factors, including depression and insomnia, among the general population in Makkah Province and Al-Madinah Province, Saudi Arabia.

**Methods:**

This analytical cross-sectional study was conducted and data were obtained through a self-administered online questionnaire using the Patient Health Questionnaire-2 (PHQ-2) for depression, the Nomophobia Questionnaire (NMP-Q), and Insomnia Severity Index (ISI).

**Results:**

A total of 1022 participants completed the questionnaire. The prevalence of nomophobia was 96.7%. Moderate nomophobia was prevalent (47.8%). Based on the PHQ-2, possible depression was identified in 47.3% of the respondents. 37.1% had sub-threshold insomnia. In terms of personal psychiatric history, the most common mental disorders in the participants included generalized anxiety disorder (9.9%) and major depressive disorder (9.7%). 61.6% of them used mobile devices for more than four hours per day.

**Conclusion:**

Nomophobia is prevalent in the Makkah and Al-Madinah provinces in Saudi Arabia. The risk of nomophobia was significantly higher for participants who spent more hours using mobile devices, those with possible depression, and those having irritable bowel syndrome.

## 1 INTRODUCTION

Nomophobia can be defined as an exaggerated and irrational fear of not having a mobile phone, and it has emerged to be a growing concern in today’s digitally connected society. The name, which was coined in 2008, is derived from the words “no mobile phone” and “phobia” [1-4]. This concept is based on individuals’ over-dependence on mobile devices to the point that they experience significant distress and anxiety when they are separated from their phones [5-7]. Nomophobia is not technically classified as a phobia and is not included in the Diagnostic and Statistical Manual of Mental Disorders (DSM-5) despite being extremely common and having a number of health consequences [2]. It has been demonstrated that individuals with co-occurring mental disorders, such as anxiety disorders and/or depressive disorders, in addition to psychological factors, such as poor self-esteem, are more likely to experience nomophobia [8-10]. Moreover, the etiology of nomophobia has been linked to many factors, including young age, female gender, and some personality traits, such as neuroticism and extraversion [6, 11, 12]. In the current digital age, nomophobia is a widespread public health issue, and it is associated with a range of negative consequences, including reduced psychological well-being, sleep disturbances, and impaired social functioning [6, 10, 11, 13]. Although the direct financial costs of nomophobia have not been fully quantified, this condition can lead to significant morbidity and potential mortality. The risks associated with nomophobia, such as increased accident rates due to device-related distraction, underscore the need for greater awareness and intervention [13]. Several studies have been conducted on nomophobia in different countries and among different age groups using different methods and considering various factors. For example, a study involving the general populations of Saudi Arabia and Jordan showed nomophobia to be especially prevalent (51.2%) among adults under 30 years of age [6]. Another Saudi study reported a prevalence of moderate nomophobia among respiratory therapy students of 97.3%. Female and non-smoking students were particularly affected, achieving average grade point averages lower than 3.49 out of 5 [14]. The prevalence of severe nomophobia in Deaf/Hard-of-Hearing (DHH) individuals in Saudi Arabia was reported to be 71.6% [15]. Similarly, a study on young people in Turkey found 20% of the youth to have mild nomophobia, 71.5% moderate nomophobia, and 8.5% severe nomophobia [16]. Another study on undergraduate students in Pakistan revealed that 40.8% had severe nomophobia, 48.5% had moderate nomophobia, and 10.5% had mild nomophobia [17]. Given these alarming findings, we sought to measure the prevalence of nomophobia and its relation to various psychological factors, depression, and insomnia among the general population in Makkah Province and Al-Madinah Province, Saudi Arabia. However, it is important to mention that previously conducted studies have involved many limitations. Some studies have fully depended only on web-based online surveying or used self-administered questionnaires, both online and on paper. Also, some studies have targeted specific demographic groups rather than the overall general population, such as a specific age group of people, people diagnosed with disabilities, or college/university students [6, 14-18]. Additionally, none of those studies has aimed to establish the relationship among nomophobia, insomnia, and depression.

## 2 MATERIALS AND METHODS

### 2.1 Study Design, Setting, and Participants

This study was a web-based analytical cross-sectional study. Data were obtained through an online questionnaire targeting the population of Makkah Province and Al-Madinah Province, Saudi Arabia, from July to October 2023 through social media platforms, like Facebook, WhatsApp, Instagram, and X platform. The inclusion criteria were adults aged 18 years old and older who lived in any city in these two provinces in Saudi Arabia. The participants were recruited using a convenience sampling approach. The Makkah and Madinah Provinces have a high population density in the Kingdom of Saudi Arabia [19].

### 2.2 Questionnaire

An online Arabic questionnaire was created using Google Forms. Respondents received electronic links that included the research objectives, target population, and a request to participate voluntarily. The questionnaire included four sections, encompassing demographic data, the Patient Health Questionnaire-2 (PHQ-2), the Nomophobia Questionnaire (NMP-Q), and the Insomnia Severity Index (ISI).

The first section collected demographic data, including age, sex, nationality, marital status, level of education, employment status, province, city, personal income, family income, personal history of mental disorders in addition to diabetes and irritable bowel syndrome, and family history of mental disorders in addition to diabetes and irritable bowel syndrome. We also asked the participants about the duration of daily smartphone usage for leisure activities (Less than 1 hour, 1-2 hours, 2-3 hours, 3-4 hours, or More than 4 hours).

The second section included the PHQ-2, which was developed by Kroenke and colleagues [20]. It assesses the frequency of low mood and anhedonia in the previous two weeks and includes the first two components of the PHQ-9. The PHQ-2 is designed to screen for depression in a “first-step” manner. Patients screened positive should be assessed further with the PHQ-9 to see if they satisfy the criteria for a depressive illness. PHQ-2 scores range between 0 and 6. The best cutoff criterion for screening for depression with the PHQ-2 is a score of 3. Major depressive disorder is likely present if the score is 3 or higher. The sensitivity and specificity (95% CI) of the PHQ-2 for cutoff scores of 3 or higher were found to be 0.72 (0.67–0.77) and 0.85 (0.83–0.87), respectively. It has been shown that the Arabic version of the PHQ is a reliable and valid instrument for screening a Saudi Arabian sample for depression, anxiety, somatic disorders, and panic disorders [21].

The third section included the NMP-Q to assess nomophobia. The NMP-Q was developed by Yildirim and Correia in 2015 [5] and consists of 20 questions, each of which is assessed on a seven-point Likert scale. The total NMP-Q score ranges from 20 to 140. The interpretation of the NMP-Q score into the level of nomophobia is shown in Table **1**. NMP-Q consists of four factors: factor 1: inability to communicate; factor 2: loss of connectivity; factor 3: inability to acquire information; and factor 4: sacrifice of convenience. The internal validity and reliability of the NMP-Q dimensions are considered good (0.78–0.92). Exploratory factor analysis indicated that the NMP-Q has a four-factor structure that correlates to the characteristics of nomophobia [5]. The questionnaire has been validated in the Arabic language, exhibiting satisfactory psychometric properties, with a Cronbach’s alpha coefficient of 0.879 [22]. In this study, we used the Arabic version of the measure, available from the Saudi Ministry of Health’s website [23].

The fourth section focused on insomnia using the Insomnia Severity Index (ISI) to identify cases of adult insomnia over the past month [24, 25]. A five-point scale was employed in the assessment of insomnia severity, with response options ranging from 0 (no problem) to 4 (a very severe problem) for all items. An aggregate score was calculated, ranging from 0 to 28. The interpretation of the ISI score into the level of insomina is shown in Table **2****.** In this study, the validated Arabic version of the scale was used, which has demonstrated good psychometric properties [26].

### 2.3 Sample Size Calculation

The minimum sample size necessary for this research was computed using the OpenEpi version 3.0, considering the following factors: a Confidence Interval (CI) of 95%; an anticipated percentage of frequency of 50%; and setting the design effect as 1, and that the population size of Makkah and Al-Madinah Provinces combined is approximately 10,159,446 according to the General Authority [27]. The sample size was estimated to be 385 participants. To allow for possible data loss, the total required sample size was 450 participants. The survey link was available to accept responses during the data collection period and it was re-posted weekly through social media channels to increase response. The response rate was high and the required sample size was achieved before the data collection period ended. During the process of data cleaning, participants with missing data were eliminated, and the total number of participants after elimination was 1022.

### 2.4 Statistical Analysis

Data were analyzed using RStudio (R version 4.3.0). The data variables have been presented as frequencies and percentages. The association between the participants’ characteristics, such as sociodemographic and clinical characteristics, including personal and family history, and nomophobia or insomnia was assessed using inferential analysis tests, including a Pearson’s Chi-squared test or a Fisher’s exact test whenever applicable. Risk factors for having moderate-to-severe nomophobia or moderate-to-severe insomnia were assessed using a binary logistic regression analysis with each outcome in a separate model. The variables that were significantly associated based on the inferential analysis were entered into the multivariable models as independent variables. The results have been expressed as Odds Ratios (ORs) and 95% Confidence Intervals (95% CIs). A p-value < 0.05 indicated statistical significance.

## 3 RESULTS

### 3.1 Sociodemographic and Clinical Characteristics

In the current study, we analyzed data from 1022 participants. The majority of the participants were in the age range of 18 to less than 30 years (79.7%), were of Saudi nationality (88.5%), and were female (75.0%). Regarding marital status, a significant proportion were single (74.8%), and in terms of education, the highest proportion had completed college or university education (58.9%). A substantial number of the participants were identified as students (64.3%) in terms of employment status, and the most prevalent province was Makkah Province (63.7%). Additionally, the majority of participants reported a personal income of less than 5000 SAR per month (80.9%), while 44.6% had a family income within the range of 5000 to 15,000 SAR per month. Based on the PHQ-2 questionnaire, the likelihood of major depressive disorder was identified among 47.3% of the respondents. In terms of personal psychiatric history, the most common mental disorders the participants were diagnosed with by a physician or psychiatrist included generalized anxiety disorder (9.9%) and major depressive disorder (9.7%), while a confirmed family history of these conditions was reported by 10.2% and 14.3% of the respondents, respectively. Diabetes and irritable bowel syndrome were prevalent among 3.3% and 15.0% of participants and among 43.5% and 33.2% of participants’ families, respectively (Table **3**).

### 3.2 Frequency of Mobile Device Usage per Day and Characteristics of Nomophobia

**Fig. (1):**
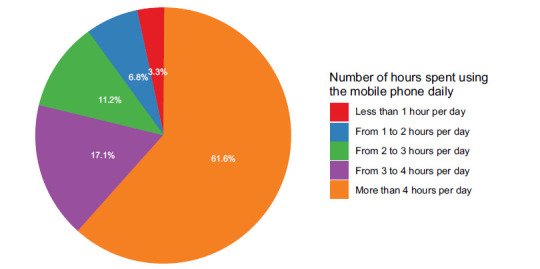
Frequency of mobile use per day.

**Fig. (2):**
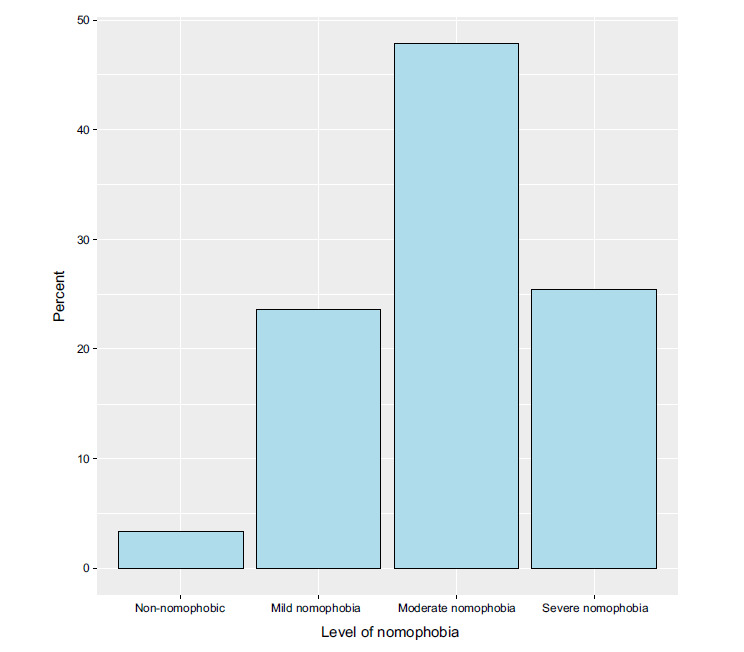
The participants’ levels of nomophobia.

Only 10.1% of the respondents indicated that they use their mobile phones two hours or less per day, whereas 61.6% of them used mobile devices more than four hours per day (Fig. **1**). Notably, only 3.2% of the respondents were not affected by nomophobia, and 23.6% of them had mild nomophobia. Moderate and severe nomophobia were prevalent among 47.8% and 25.3% of the respondents, respectively (Fig. **2**).

### 3.3 The Association between Sociodemographic Characteristics and Nomophobia and Insomnia

The inferential analysis revealed a statistically significant association between the number of hours spent using a phone daily and the severity of nomophobia (p < 0.001). Among those spending less than one hour per day, a majority exhibited “no/mild nomophobia” (76.5%), whereas a substantial proportion of participants spending more than four hours per day on their phones exhibited “moderate/severe nomophobia” (82.1%; Table **4**).

**Fig. (3):**
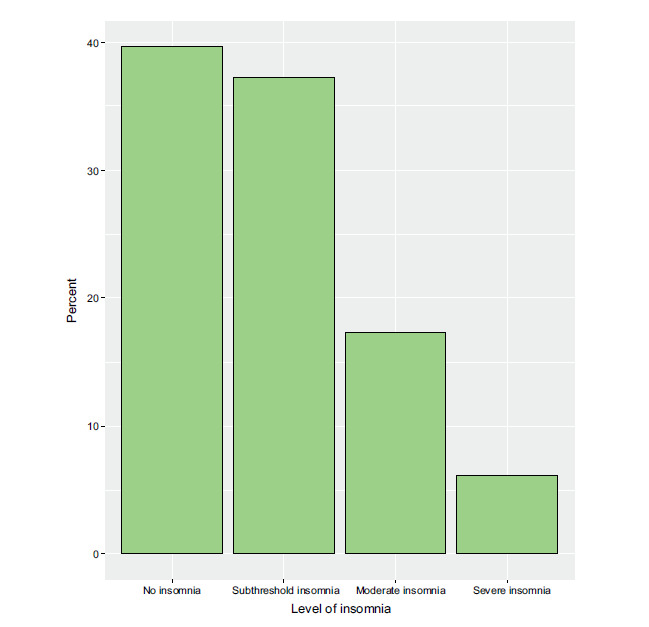
Participants’ levels of insomnia.

More than one-third of the respondents had sub-threshold insomnia (37.1%), whereas 17.2% had moderate insomnia and 6.0% had severe insomnia (Fig. **3**). Notably, the proportion of individuals with “moderate/severe insomnia” varied significantly by age (*p* = 0.013), with a higher prevalence observed among participants aged 18 to <30 years (25.3%) compared to those in older age groups. The province of residence also showed a significant association with insomnia (*p* = 0.009), as a higher proportion of individuals in Al-Madinah Province (27.8%) experienced “moderate/severe insomnia” compared to individuals in Makkah Province (20.6%). Importantly, the presence of nomophobia (*p* < 0.001) and the number of hours spent using a phone daily (*p* < 0.001) were both strongly associated with the severity of insomnia. Specifically, participants with “moderate/severe nomo- phobia” (27.9%) and those spending more than four hours per day using their phones (28.3%) had significantly higher proportions of “moderate/severe insomnia” (Table **4**).

### 3.4 The Association between Clinical Characteristics and Nomophobia and Insomnia

A substantial and statistically significant association was observed between the presence of depression, as indicated by a PHQ-2 score ≥ 3, and the severity of nomophobia (p < 0.001). Notably, 82.0% of participants with depression exhibited “moderate/severe nomophobia,” while 65.3% of those without depression reported the same. Likewise, a significantly higher proportion of participants with a previously confirmed diagnosis of depression had moderate-to-severe nomophobia (81.8%) compared to those without depression (72.3%, p = 0.041). Additionally, there was a statistically significant association between irritable bowel syndrome and the severity of nomophobia (p = 0.029), with 80.4% of individuals with irritable bowel syndrome reporting “moderate/severe nomophobia” compared to 71.9% of those without the condition (Table **5**).

Furthermore, the presence of depression, as indicated by a PHQ-2 score ≥ 3, was strongly associated with the presence of “moderate/severe insomnia” (*p* < 0.001). Among those with depression, 36.4% experienced “moderate/severe insomnia,” while only 11.3% of individuals without depression reported the same. Additionally, generalized anxiety disorder was significantly associated with insomnia (*p* = 0.002), with 35.6% of individuals with the disorder experiencing “moderate/severe insomnia” compared to 21.8% of those without it. Panic disorder was also significantly associated with insomnia (*p* = 0.008), as 42.4% of participants with panic disorder exhibited “moderate/severe insomnia,” while 22.5% of those without the disorder reported the same. Furthermore, several familial factors were significantly associated with insomnia, including familial major depressive disorder (*p* = 0.001), familial panic disorder (*p* = 0.045), familial generalized anxiety disorder (*p* = 0.029), familial irritable bowel syndrome (*p* = 0.035), and familial diabetes mellitus (*p* < 0.001; Table **5**).

### 3.5 Risk Factors for Nomophobia

The variables that were significantly associated with nomophobia in the inferential analysis were used as independent variables in the multivariable logistic regression model; however, since we had two measures of depression based on PHQ-2 and a previously confirmed diagnosis, we sought to incorporate PHQ-2-based depression in the model to avoid the risk of multicollinearity of the same condition. The results revealed that as the daily duration of mobile phone use increased, the risk of nomophobia significantly escalated, with ORs of 4.13 (95% CI: 1.76–10.6, *p* = 0.002) for two to three hours per day, 8.62 (95% CI: 3.74–21.9, *p* < 0.001) for three to four hours per day, and 13.0 (95% CI: 5.89–31.7, *p* < 0.001) for more than four hours per day. Additionally, participants with depression, as indicated by a PHQ-2 score ≥3, exhibited a significantly increased risk of nomophobia, with an OR of 1.99 (95% CI: 1.46–2.72, *p* < 0.001). Furthermore, individuals with irritable bowel syndrome had a significantly elevated risk of nomophobia, with an OR of 1.62 (95% CI: 1.04–2.60, *p* = 0.037; Table **6**).

### 3.6 Risk Factors for Insomnia

Participants with “moderate/severe nomophobia” had significantly higher odds of experiencing insomnia (OR = 2.60, 95% CI: 1.66–4.18, *p* < 0.001) compared to those with “no/mild nomophobia.” Similarly, individuals with depression, as indicated by a PHQ-2 score ≥3, exhibited substantially increased odds of insomnia (OR = 3.49, 95% CI: 2.50–4.93, *p* < 0.001) compared to those without depression. Furthermore, the presence of familial diabetes mellitus was associated with a higher likelihood of experiencing insomnia (OR = 1.57, 95% CI: 1.12–2.21, *p* = 0.010). Finally, residing in Al-Madinah Province was found to be a significant risk factor for insomnia (OR = 1.55, 95% CI: 1.12–2.13, *p* = 0.008) compared to Makkah Province (Table **7**).

## 4 DISCUSSION

This study has examined the prevalence of nomophobia in Saudi Arabia. Specifically, this study has investigated the following aspects: (1) the association between nomophobia and frequency of mobile device usage per day; (2) the association between sociodemographic characteristics and nomophobia and insomnia; (3) the association between clinical characteristics and nomophobia and insomnia.

Based on the NMP-Q, the prevalence of nomophobia in our study was 96.7%. The participants exhibited varying degrees of nomophobia, with 23.6% classified as having mild nomophobia, 47.8% as having moderate nomophobia, and 25.3% as having severe nomophobia. These results highlight the substantial prevalence of nomophobia in the sample, emphasizing the psychological impact of excessive mobile phone use. This result is consistent with previous studies using the NMP-Q. For example, a study on a sample of respiratory therapy students found 97.3% to have nomophobia [14]. Another study involving Saudi medical students in Makkah, Saudi Arabia, showed that 99% of them had nomophobia [28]. However, our results were higher than those in studies that used the International Classification of Diseases 10th Revision (ICD-10) substance dependency syndrome criteria, which is a diagnostic questionnaire consisting of 6 items [29]. In an observational cross-sectional study conducted in Saudi Arabia and Jordan, 51.2% of the 5191 participants from the general population were diagnosed with nomophobia [6]. Another study on university students in five Arab countries, including Egypt, Saudi Arabia, Jordan, Lebanon, and Bahrain, reported that 55.6% of the sample met the criteria of nomophobia [18].

Regarding the association between the severity of nomophobia and the frequency of using mobile devices, results from our study have indicated a relationship between the frequency of mobile use and participants’ level of nomophobia. That is, most participants were classified as having a “moderate” level of nomophobia, followed by a “severe” level of nomophobia, while only a small number of participants had an “absence” level of nomophobia. Results from this study align with previous studies that have focused on exploring the relationship between mobile use and its association with increased levels of nomophobia. Our results have indicated a significant correlation between increased duration of mobile phone use and nomophobia, consistent with another study showing high mobile phone use to be a strong predictor of nomophobia [30, 31]. Furthermore, other studies have shed light on the prevalence of nomophobia among various populations. For instance, a study in India [32] found that the prevalence of nomophobia was higher among female participants (49.6%) than among males (37.1%), indicating a potential gender difference in the manifestation of nomophobia. This association was found to be statistically significant (p = 0.027). Additionally, the study revealed that first-year professionals had the highest prevalence of nomophobia (59.9%), while interns had the lowest prevalence (17.5%; p = 0.000). Similarly, an investigation conducted in Oman [33] assessed nomophobia in a sample of 740 students, comprising an equal number of women and men, and found a remarkably high prevalence of 99.33%. The mean score across participants was 82.90, indicating a moderate level of nomophobia on average. The results revealed that 20% of students had mild nomophobia, 15% had severe nomophobia, and the majority (65%) had moderate nomophobia. The results from these studies indicate that nomophobia is a widespread issue, particularly among younger individuals, females, and those with higher levels of education. The comparison of different studies underscores the importance of considering cultural and gender factors in understanding nomophobia. These research findings can contribute to raising awareness about the detrimental effects of excessive mobile phone use and inform the development of interventions to mitigate the impact of nomophobia on individuals’ well-being.

Furthermore, the findings of the current study related to the association among nomophobia, sociodemographic characteristics, and clinical characteristics have indicated participants with depression to show “moderate/severe nomophobia.” Similarly, findings from one study [34] that investigated the correlation between nomophobia and depression in adolescents indicated a significant association between nomophobia and adolescents with depression. Results from the current study and a previous study [34] support the fact that depression is a risk factor despite age being a variable. Furthermore, individuals with irritable bowel syndrome were also found to have a higher likelihood of experiencing increased nomophobia severity. Similarly, there was a significant correlation between nomophobia and depression, as indicated by a PHQ-2 score ≥ 3, in line with results reported in other studies [10, 34].

The risk factors associated with insomnia in this study were found to be moderate/severe nomophobia, familial diabetes mellitus, residing in Al-Madinah Province, and depression. The results revealed a significant correlation between moderate/severe nomophobia and insomnia, consistent with a study showing excessive use of smartphones as related to lower sleep quality [35]. Additionally, in our study, familial diabetes mellitus was found to be significantly associated with a higher likelihood of experiencing insomnia despite the outcome of this research, which found no significant correlation between diabetes mellitus and insomnia [36]. Additionally, residing in Al-Madinah Province was found to be a significant risk factor for insomnia compared to residing in Makkah Province. Furthermore, the results of this study have indicated a significant correlation between insomnia and depression, as indicated by a PHQ-2 score ≥ 3. This result supports the findings of a previous study showing that sleep disturbance is the most common symptom in patients with depression [37]. However, there was no significant correlation between irritable bowel syndrome and insomnia, in contrast to a study that reported a higher prevalence of sleep disorders among individuals with irritable bowel syndrome [38]. The difference in these results may be due to the different areas, ages, and occupations considered in the two studies.

The strengths of this study included the large sample size and the three Arabic-validated instruments employed to address the research topic. In terms of limitations, the study utilized a cross-sectional design, and thus causality could not be established. Further, the study used an online self-administered convenience sample, which is unlikely to be representative of the population and could cause selection bias. Recall bias may have been an issue due to the use of self-reports for data collection, and questionnaires were used that focused solely on symptoms rather than diagnoses.

## CONCLUSION

Nomophobia was found to be prevalent in Makkah and Al-Madinah Provinces in Saudi Arabia. The risk of nomophobia was significantly higher for participants who spent more hours using their mobile devices, those with possible depression (PHQ-2 score ≥ 3), and those with irritable bowel syndrome. More than one-third of the respondents had sub-threshold insomnia, 17.2% had moderate insomnia, and 6.0% had severe insomnia. Finally, individuals with moderate/severe nomophobia, depression, familial diabetes mellitus, and those residing in Al-Madinah Province were significantly more likely to experience insomnia.

## Figures and Tables

**Table 1:** The interpretation of the NMP-Q score into the level of nomophobia.

**Score**	**Nomophobia Level**
NMP-Q score= 20	Absent
21 ≤ NMP-Q score = < 60	Mild
60 ≤ NMP-Q score = < 100	Moderate
100 ≤ NMP-Q score = < 140	Severe

**Table 2:** The interpretation of the ISI score into the level of insomnia.

**Score**	**Level of Insomnia**
0 - 7	No clinically significant insomnia
8 - 14	Subthreshold
15 - 21	Clinical insomnia (moderate severity)
22 - 28	Clinical insomnia (severe)

**Table 3:** Sociodemographic and clinical characteristics of the participants.

Characteristic	N = 1022
**Age (years)**	-
18 to <30	815 (79.7%)
30 to <45	133 (13.0%)
45 to <60	58 (5.7%)
60 or more	16 (1.6%)
**Nationality**	-
Saudi	904 (88.5%)
Non-Saudi	118 (11.5%)
**Sex**	-
Male	256 (25.0%)
Female	766 (75.0%)
**Marital status**	-
Single	764 (74.8%)
Married	233 (22.8%)
Divorced	23 (2.3%)
Widow	2 (0.2%)
**Level of education**	-
Elementary school	6 (0.6%)
Middle school	11 (1.1%)
High school	328 (32.1%)
Diploma	39 (3.8%)
College/University	602 (58.9%)
Master’s	29 (2.8%)
Doctorate	7 (0.7%)
**Employment status**	-
Unemployed	67 (6.6%)
Student	657 (64.3%)
Employee in the government sector	74 (7.2%)
Employee in the private sector	65 (6.4%)
Employee in the government medical sector	28 (2.7%)
Employee in the private medical sector	3 (0.3%)
Employee in the military sector	6 (0.6%)
Stay-at-home	97 (9.5%)
Retired	25 (2.4%)
**Province**	-
Al-Madinah Province	371 (36.3%)
Makkah Province	651 (63.7%)
**City**	-
Al-Madinah Al-Munawwarah	331 (32.4%)
Jeddah	283 (27.7%)
Makkah Al-Mukarramah	255 (25.0%)
Al-Taif	84 (8.2%)
Others	69 (6.8%)
**Personal income (SAR)**	-
Less than 5000 SAR per month	827 (80.9%)
From 5000 SAR to 15000 SAR per month	141 (13.8%)
More than 15000 SAR per month	54 (5.3%)
**Family income (SAR)**	-
Less than 5000 SAR per month	196 (19.2%)
From 5000 SAR to 15000 SAR per month	456 (44.6%)
More than 15000 SAR per month	370 (36.2%)
**Major depressive disorder (PHQ-2 score ≥ 3)**	-
No depression	539 (52.7%)
Depression	483 (47.3%)
**Personal history**	-
Major depressive disorder	99 (9.7%)
Bipolar affective disorder	8 (0.8%)
Panic disorder	33 (3.2%)
Generalized anxiety disorder	101 (9.9%)
Postpartum depression	15 (1.5%)
Postpartum psychosis	3 (0.3%)
Schizophrenia	2 (0.2%)
Irritable bowel syndrome	153 (15.0%)
Diabetes mellitus	34 (3.3%)
**Family history**	-
Familial major depressive disorder	146 (14.3%)
Familial bipolar affective disorder	47 (4.6%)
Familial panic disorder	59 (5.8%)
Familial generalized anxiety disorder	104 (10.2%)
Familial postpartum depression	69 (6.8%)
Familial postpartum psychosis	5 (0.5%)
Familial schizophrenia	25 (2.4%)
Familial irritable bowel syndrome	339 (33.2%)
Familial diabetes mellitus	445 (43.5%)

**Table 4:** The association between sociodemographic characteristics and nomophobia and insomnia.

Characteristic	No/mild Nomophobia (N = 274)	Moderate/severe Nomophobia (N = 748)	p-value	No/sub-threshold Insomnia (N = 785)	Moderate/severe Insomnia (N = 237)	p-value
Age (years)	-	-	0.618	-	-	0.013
18 to <30	213 (26.1%)	602 (73.9%)	-	609 (74.7%)	206 (25.3%)	-
30 to <45	39 (29.3%)	94 (70.7%)	-	112 (84.2%)	21 (15.8%)	-
45 to <60	16 (27.6%)	42 (72.4%)	-	49 (84.5%)	9 (15.5%)	-
60 or more	6 (37.5%)	10 (62.5%)	-	15 (93.8%)	1 (6.3%)	-
Nationality	-	-	0.092	-	-	0.213
Saudi	250 (27.7%)	654 (72.3%)	-	689 (76.2%)	215 (23.8%)	-
Non-Saudi	24 (20.3%)	94 (79.7%)	-	96 (81.4%)	22 (18.6%)	-
Sex	-	-	0.477	-	-	0.686
Male	73 (28.5%)	183 (71.5%)	-	199 (77.7%)	57 (22.3%)	-
Female	201 (26.2%)	565 (73.8%)	-	586 (76.5%)	180 (23.5%)	-
Marital status	-	-	0.844	-	-	0.182
Single	204 (26.7%)	560 (73.3%)	-	577 (75.5%)	187 (24.5%)	-
Married	63 (27.0%)	170 (73.0%)	-	187 (80.3%)	46 (19.7%)	-
Divorced	6 (26.1%)	17 (73.9%)	-	20 (87.0%)	3 (13.0%)	-
Widow	1 (50.0%)	1 (50.0%)	-	1 (50.0%)	1 (50.0%)	-
Level of education	-	-	0.750	-	-	0.559
Elementary school	2 (33.3%)	4 (66.7%)	-	6 (100.0%)	0 (0.0%)	-
Middle school	4 (36.4%)	7 (63.6%)	-	10 (90.9%)	1 (9.1%)	-
High school	94 (28.7%)	234 (71.3%)	-	253 (77.1%)	75 (22.9%)	-
Diploma	10 (25.6%)	29 (74.4%)	-	33 (84.6%)	6 (15.4%)	-
College/University	152 (25.2%)	450 (74.8%)	-	453 (75.2%)	149 (24.8%)	-
Master’s	10 (34.5%)	19 (65.5%)	-	24 (82.8%)	5 (17.2%)	-
Doctorate	2 (28.6%)	5 (71.4%)	-	6 (85.7%)	1 (14.3%)	-
Employment status	-	-	0.222	-	-	0.122
Unemployed	21 (31.3%)	46 (68.7%)	-	44 (65.7%)	23 (34.3%)	-
Student	171 (26.0%)	486 (74.0%)	-	496 (75.5%)	161 (24.5%)	-
Employee in the government sector	18 (24.3%)	56 (75.7%)	-	62 (83.8%)	12 (16.2%)	-
Employee in the private sector	17 (26.2%)	48 (73.8%)	-	49 (75.4%)	16 (24.6%)	-
Employee in the government medical sector	7 (25.0%)	21 (75.0%)	-	24 (85.7%)	4 (14.3%)	-
Employee in the private medical sector	0 (0.0%)	3 (100.0%)	-	3 (100.0%)	0 (0.0%)	-
Employee in the military sector	5 (83.3%)	1 (16.7%)	-	6 (100.0%)	0 (0.0%)	-
Stay-at-home	28 (28.9%)	69 (71.1%)	-	80 (82.5%)	17 (17.5%)	-
Retired	7 (28.0%)	18 (72.0%)	-	21 (84.0%)	4 (16.0%)	-
Province	-	-	0.122	-	-	0.009
Al-Madinah Province	110 (29.6%)	261 (70.4%)	-	268 (72.2%)	103 (27.8%)	-
Makkah Province	164 (25.2%)	487 (74.8%)	-	517 (79.4%)	134 (20.6%)	-
City	-	-	0.177	-	-	0.102
Al-Madinah Al-Munawwarah	101 (30.5%)	230 (69.5%)	-	240 (72.5%)	91 (27.5%)	-
Jeddah	79 (27.9%)	204 (72.1%)	-	222 (78.4%)	61 (21.6%)	-
Makkah Al-Mukarramah	61 (23.9%)	194 (76.1%)	-	203 (79.6%)	52 (20.4%)	-
Al-Taif	16 (19.0%)	68 (81.0%)	-	70 (83.3%)	14 (16.7%)	-
Others	17 (24.6%)	52 (75.4%)	-	50 (72.5%)	19 (27.5%)	-
Personal income (SAR)	-	-	0.390	-	-	0.051
Less than 5000 SAR per month	225 (27.2%)	602 (72.8%)	-	623 (75.3%)	204 (24.7%)	-
From 5000 SAR to 15,000 SAR per month	32 (22.7%)	109 (77.3%)	-	115 (81.6%)	26 (18.4%)	-
More than 15,000 SAR per month	17 (31.5%)	37 (68.5%)	-	47 (87.0%)	7 (13.0%)	-
Family income (SAR)	-	-	0.228	-	-	0.391
Less than 5000 SAR per month	60 (30.6%)	136 (69.4%)	-	147 (75.0%)	49 (25.0%)	-
From 5000 SAR to 15,000 SAR per month	125 (27.4%)	331 (72.6%)	-	345 (75.7%)	111 (24.3%)	-
More than 15,000 SAR per month	89 (24.1%)	281 (75.9%)	-	293 (79.2%)	77 (20.8%)	-
How many hours do you spend using your phone daily?	-	-	<0.001	-	-	<0.001
Less than 1 h per day	26 (76.5%)	8 (23.5%)	-	31 (91.2%)	3 (8.8%)	-
From 1 to 2 h per day	40 (58.0%)	29 (42.0%)	-	61 (88.4%)	8 (11.6%)	-
From 2 to 3 h per day	49 (43.0%)	65 (57.0%)	-	94 (82.5%)	20 (17.5%)	-
From 3 to 4 h per day	46 (26.3%)	129 (73.7%)	-	147 (84.0%)	28 (16.0%)	-
More than 4 h per day	113 (17.9%)	517 (82.1%)	-	452 (71.7%)	178 (28.3%)	-
Nomophobia	-	-	-	-	-	<0.001
No/mild nomophobia	-	-	-	246 (89.8%)	28 (10.2%)	-
Moderate/severe nomophobia	-	-	-	539 (72.1%)	209 (27.9%)	-

**Table 5:** The association between clinical characteristics and nomophobia and insomnia.

Characteristic	No/mild Nomophobia (N = 274)	Moderate/severe Nomophobia (N = 748)	*p*-value	No/sub-threshold Insomnia (N = 785)	Moderate/severe Insomnia (N = 237)	p-value
Major depressive disorder (PHQ-2 score ≥ 3)	-	-	<0.001	-	-	<0.001
No depression	187 (34.7%)	352 (65.3%)	-	478 (88.7%)	61 (11.3%)	-
Depression	87 (18.0%)	396 (82.0%)	-	307 (63.6%)	176 (36.4%)	-
Major depressive disorder	-	-	0.041	-	-	<0.001
No	256 (27.7%)	667 (72.3%)	-	723 (78.3%)	200 (21.7%)	-
Yes	18 (18.2%)	81 (81.8%)	-	62 (62.6%)	37 (37.4%)	-
Bipolar affective disorder	-	-	0.689	-	-	0.089
No	273 (26.9%)	741 (73.1%)	-	781 (77.0%)	233 (23.0%)	-
Yes	1 (12.5%)	7 (87.5%)	-	4 (50.0%)	4 (50.0%)	-
Panic disorder	-	-	0.053	-	-	0.008
No	270 (27.3%)	719 (72.7%)	-	766 (77.5%)	223 (22.5%)	-
Yes	4 (12.1%)	29 (87.9%)	-	19 (57.6%)	14 (42.4%)	-
Generalized anxiety disorder	-	-	0.094	-	-	0.002
No	254 (27.6%)	667 (72.4%)	-	720 (78.2%)	201 (21.8%)	-
Yes	20 (19.8%)	81 (80.2%)	-	65 (64.4%)	36 (35.6%)	-
Postpartum depression	-	-	>0.999	-	-	0.358
No	270 (26.8%)	737 (73.2%)	-	775 (77.0%)	232 (23.0%)	-
Yes	4 (26.7%)	11 (73.3%)	-	10 (66.7%)	5 (33.3%)	-
Postpartum psychosis	-	-	0.568	-	-	0.136
No	274 (26.9%)	745 (73.1%)	-	784 (76.9%)	235 (23.1%)	-
Yes	0 (0.0%)	3 (100.0%)	-	1 (33.3%)	2 (66.7%)	-
Schizophrenia	-	-	>0.999	-	-	0.054
No	274 (26.9%)	746 (73.1%)	-	785 (77.0%)	235 (23.0%)	-
Yes	0 (0.0%)	2 (100.0%)	-	0 (0.0%)	2 (100.0%)	-
Irritable bowel syndrome	-	-	0.029	-	-	0.176
No	244 (28.1%)	625 (71.9%)	-	674 (77.6%)	195 (22.4%)	-
Yes	30 (19.6%)	123 (80.4%)	-	111 (72.5%)	42 (27.5%)	-
Diabetes mellitus	-	-	0.458	-	-	0.382
No	263 (26.6%)	725 (73.4%)	-	761 (77.0%)	227 (23.0%)	-
Yes	11 (32.4%)	23 (67.6%)	-	24 (70.6%)	10 (29.4%)	-
Familial major depressive disorder	-	-	0.215	-	-	0.001
No	241 (27.5%)	635 (72.5%)	-	688 (78.5%)	188 (21.5%)	-
Yes	33 (22.6%)	113 (77.4%)	-	97 (66.4%)	49 (33.6%)	-
Familial bipolar affective disorder	-	-	0.589	-	-	0.147
No	263 (27.0%)	712 (73.0%)	-	753 (77.2%)	222 (22.8%)	-
Yes	11 (23.4%)	36 (76.6%)	-	32 (68.1%)	15 (31.9%)	-
Familial panic disorder	-	-	0.145	-	-	0.045
No	263 (27.3%)	700 (72.7%)	-	746 (77.5%)	217 (22.5%)	-
Yes	11 (18.6%)	48 (81.4%)	-	39 (66.1%)	20 (33.9%)	-
Familial generalized anxiety disorder	-	-	0.108	-	-	0.029
No	253 (27.6%)	665 (72.4%)	-	714 (77.8%)	204 (22.2%)	-
Yes	21 (20.2%)	83 (79.8%)	-	71 (68.3%)	33 (31.7%)	-
Familial postpartum depression	-	-	0.888	-	-	0.140
No	255 (26.8%)	698 (73.2%)	-	737 (77.3%)	216 (22.7%)	-
Yes	19 (27.5%)	50 (72.5%)	-	48 (69.6%)	21 (30.4%)	-
Familial postpartum psychosis	-	-	0.332	-	-	0.085
No	274 (26.9%)	743 (73.1%)	-	783 (77.0%)	234 (23.0%)	-
Yes	0 (0.0%)	5 (100.0%)	-	2 (40.0%)	3 (60.0%)	-
Familial schizophrenia	-	-	0.217	-	-	0.124
No	270 (27.1%)	727 (72.9%)	-	769 (77.1%)	228 (22.9%)	-
Yes	4 (16.0%)	21 (84.0%)	-	16 (64.0%)	9 (36.0%)	-
Familial irritable bowel syndrome	-	-	0.183	-	-	0.035
No	192 (28.1%)	491 (71.9%)	-	538 (78.8%)	145 (21.2%)	-
Yes	82 (24.2%)	257 (75.8%)	-	247 (72.9%)	92 (27.1%)	-
Familial diabetes mellitus	-	-	0.080	-	-	<0.001
No	167 (28.9%)	410 (71.1%)	-	469 (81.3%)	108 (18.7%)	-
Yes	107 (24.0%)	338 (76.0%)	-	316 (71.0%)	129 (29.0%)	-

**Table 6:** Risk factors for nomophobia.

**Characteristic**	**OR**	**95% CI**	***p*-value**
Hours spent using the mobile phone	-	-	-
Less than 1 h per day	Reference	Reference	-
From 1 to 2 h per day	2.20	0.88, 5.92	0.100
From 2 to 3 h per day	4.13	1.76, 10.6	0.002
From 3 to 4 h per day	8.62	3.74, 21.9	<0.001
More than 4 h per day	13.0	5.89, 31.7	<0.001
Major depressive disorder (PHQ-2 score ≥ 3)	-	-	-
No depression	Reference	Reference	-
Depression	1.99	1.46, 2.72	<0.001
Irritable bowel syndrome	-	-	-
No	Reference	Reference	-
Yes	1.62	1.04, 2.60	0.037

**Abbreviations: **OR = Odds Ratio, CI = Confidence Interval.

**Table 7:** Risk factors for insomnia.

**Characteristic**	**OR**	**95% CI**	***p*-value**
Age	0.99	0.97, 1.00	0.129
Province	-	-	-
Makkah Province	Reference	Reference	-
Al-Madinah Province	1.55	1.12, 2.13	0.008
How many hours do you spend using your phone daily?	-	-	-
Less than 1 h per day	Reference	Reference	-
From 1 to 2 h per day	0.96	0.23, 4.96	0.961
From 2 to 3 h per day	1.50	0.44, 7.01	0.557
From 3 to 4 h per day	1.02	0.30, 4.70	0.978
More than 4 h per day	1.75	0.56, 7.77	0.389
Nomophobia	-	-	-
No/mild nomophobia	Reference	Reference	-
Moderate/severe nomophobia	2.60	1.66, 4.18	<0.001
Major depressive disorder (PHQ-2 score ≥ 3)	-	-	-
No depression	Reference	Reference	-
Depression	3.49	2.50, 4.93	<0.001
Panic disorder	-	-	-
No	Reference	Reference	-
Yes	1.45	0.61, 3.37	0.391
Generalized anxiety disorder	-	-	-
No	Reference	Reference	-
Yes	1.36	0.78, 2.32	0.269
Familial major depressive disorder	-	-	-
No	Reference	Reference	-
Yes	1.19	0.73, 1.92	0.485
Familial panic disorder	-	-	-
No	Reference	Reference	-
Yes	1.05	0.52, 2.08	0.881
Familial generalized anxiety disorder	-	-	-
No	Reference	Reference	-
Yes	0.97	0.52, 1.77	0.926
Familial irritable bowel syndrome	-	-	-
No	Reference	Reference	-
Yes	0.93	0.65, 1.34	0.708
Familial diabetes mellitus	-	-	-
No	Reference	Reference	-
Yes	1.57	1.12, 2.21	0.010

**Abbreviations: **OR = Odds Ratio, CI = Confidence Interval.

## Data Availability

All the data related to this study is available upon request.

## LIST OF ABBREVIATIONS

PHQ-2: Patient Health Questionnaire-2
NMP-Q: Nomophobia Questionnaire
ISI: Insomnia Severity Index
DSM-5: Diagnostic and Statistical Manual of Mental Disorders

## STANDARDS OF REPORTING

STROBE guidelines were followed.
